# Inelastic scattering of electrons in water from first principles: cross sections and inelastic mean free path for use in Monte Carlo track-structure simulations of biological damage

**DOI:** 10.1098/rsos.212011

**Published:** 2022-05-18

**Authors:** Natalia E. Koval, Peter Koval, Fabiana Da Pieve, Jorge Kohanoff, Emilio Artacho, Dimitris Emfietzoglou

**Affiliations:** ^1^ CIC Nanogune BRTA, 20018 Donostia-San Sebastián, Spain; ^2^ Simune Atomistics SL, 20018 Donostia-San Sebastián, Spain; ^3^ Donostia International Physics Center DIPC, 20018 Donostia-San Sebastián, Spain; ^4^ Royal Belgian Institute for Space Aeronomy BIRA-IASB, 1180 Brussels, Belgium; ^5^ Queen’s University Belfast, Belfast BT7 1NN, UK; ^6^ Instituto de Fusion Nuclear ‘Guillermo Velarde’, Universidad Politecnica de Madrid, 28006 Madrid, Spain; ^7^ Ikerbasque, Basque Foundation for Science, 48011 Bilbao, Spain; ^8^ Theory of Condensed Matter, Cavendish Laboratory, University of Cambridge, Cambridge CB3 0HE, UK; ^9^ Medical Physics Laboratory, University of Ioannina Medical School, 45110 Ioannina, Greece

**Keywords:** radiation damage, inelastic electron scattering, water, linear response, time-dependent density functional theory, track-structure simulations

## Abstract

Modelling the inelastic scattering of electrons in water is fundamental, given their crucial role in biological damage. In Monte Carlo track-structure (MC-TS) codes used to assess biological damage, the energy loss function (ELF), from which cross sections are extracted, is derived from different semi-empirical optical models. Only recently have first *ab initio* results for the ELF and cross sections in water become available. For benchmarking purpose, in this work, we present *ab initio* linear-response time-dependent density functional theory calculations of the ELF of liquid water. We calculated the inelastic scattering cross sections, inelastic mean free paths, and electronic stopping power and compared our results with recent calculations and experimental data showing a good agreement. In addition, we provide an in-depth analysis of the contributions of different molecular orbitals, species and orbital angular momenta to the total ELF. Moreover, we present single-differential cross sections computed for each molecular orbital channel, which should prove useful for MC-TS simulations.

## 1. Introduction

The scattering of electrons in biological matter plays a crucial role in a variety of fields related to radiation-induced damage, such as ion-beam therapy and risk assessment in space radiation studies. In theoretical and often experimental studies of biological damage, liquid water is considered a model system. The initial response of biological material to radiation is determined, to a large extent, by the oscillator-strength distribution of its valence electrons, which leads primarily to the generation of electrons with energies of less than 100 eV [1,2]. This results from the shape of the differential cross section of molecules, which peaks at 20–30 eV and decreases to low values above 100 eV [3]. Evidence has accumulated throughout the years that very low-energy (less than 20 eV) electrons play a relevant role in bio-damage [4–8]. They constitute the so-called ‘track ends’ and are reported to have an increased biological effectiveness [9–11]. Experiments have indicated that electrons (or photons) with energies as low as few electron-volts can still induce double-strand breaks, possibly through a resonance mechanism [12–14].

Nowadays, several Monte Carlo track-structure (MC-TS) codes exist [15–18], able to describe the transport of electrons via an event-by-event simulation until low energy (approx. 10 eV), like norec [19], kurbuc [20], partrac [21], ritracks [22] and the open source Geant4-DNA [23,24]. The track structures in water are then overlaid onto DNA models ranging in complexity from simple cylindrical models of the DNA to a full atomistic description of human chromosomal DNA [25–29]. MC-TS codes either rely on pre-parameterized sets of cross sections [1] or use optical data models for the dielectric function of water based on the first Born approximation [30–34]. In such models, the energy loss function (ELF) in the dielectric formalism, from which other quantities like cross sections and inelastic mean free path (IMFP) are calculated, is determined at negligible momentum transfer *q* = 0 from experimental data (often optical data [35,36]). For finite momentum transfers *q* ≠ 0, the ELF is appropriately extended to the whole Bethe surface via dispersion models based on the electron gas theory [15] within the context of the random-phase approximation (RPA). The ELF is commonly described via a superposition of either normal or derivative Drude functions. The Drude model parameters associated with the height, width and position of the peaks are used as adjustable parameters determined by the fit to experimental data and are generally constrained by sum rules. Other models exist as well, based on Mermin functions [37], which partially incorporate effects beyond RPA.

By assuming that each electron interacts with the average field generated by all other electrons, the RPA accounts only for electrostatic screening. The exchange-correlation (XC) effects (due to the instantaneous Coulomb repulsion and the Pauli exclusion principle) are neglected in RPA. The Born approximation neglects, among other things, exchange effects between the incident and struck electrons. For high energies, such effects are only important for hard collisions, characterized by a large energy transfer. At low energies, however, the incident and the target electrons have similar energies and thus one expects that exchange effects will become relevant essentially for all collisions. Moreover, in the Born approximation, a first-order perturbation theory is used to describe the interaction between the projectile and the target, which is in principle not valid for low energies [38,39]. The use of approximations for the scattering parameters leads to differences in simulations of track structure [40]. The discrepancies in the inelastic scattering obtained with different extension algorithms to extrapolate optical data to finite momentum transfer can reach a factor of about two in the range 50–200 eV (and even larger at still lower energies) [41]. Recent studies have reported a potentially relevant effect of the different dielectric function implementations on ionization clustering [42] and DNA damage induction [43].

There is a high degree of uncertainty in the low-energy range in MC-TS codes as the cross sections become sensitive to the details of the electronic structure of the target [44–47]. Even though atomic structures are implemented in some TS codes [48–50], the absence of electronic effects and often occurring lack of reference cross sections for benchmarking make it difficult to extend the applicability of such codes to the targets other than homogeneous liquid water. In fact, codes like Geant4-DNA use the cross sections for water independently of the actual medium, only re-scaling the density.

Several recent works have been performed to ameliorate the description of the dielectric function and the IMFP of water. One way is to include the XC effects beyond RPA on the basis of the electron gas model [39,51]. Other works additionally tried improving the effects beyond the first Born approximation [29,52], improving previous dispersion algorithms [53], developing new TS codes [54,55] and clarifying differences in inelastic scattering between different condensed phases [56]. Others focus on extending the set of cross sections for electron scattering in targets other than water via pre-parameterized models [57] or multi-channel and R-matrix approaches [58].

Overall, the accuracy of the semi-empirical results for water at energies below 100 eV remains questionable. *Ab initio* calculations can provide insightful results for the dielectric function, the electron ELF and the IMFP in the whole energy range. First-principles methods do not rely on any free parameters and thus have predictive power and can be extended to a variety of targets. Nowadays, time-dependent density functional theory (TDDFT) [59,60] is the method of choice for the study of excited states, since it allows for the affordable extraction of physical information without *a priori* assumptions on the system and on the knowledge of associated cross sections. TDDFT can be formulated either in the perturbative regime [61] or via an explicit solution of the time-dependent Kohn–Sham (KS) equations [62,63] by propagating the KS orbitals in real time. Nevertheless, *ab initio* studies on the ELF and IMFP in water for low-energy electrons are limited so far. A previous real-time TDDFT study by Tavernelli [64] presented a dielectric constant of liquid water (optical limit only) with two prominent peaks as opposed to only one main peak in the experimental data [41]. A more recent work by Taioli *et al.* [65] presented linear-response TDDFT (LR-TDDFT) calculations of the ELF for liquid water. The sample of 32 molecules was obtained from a larger sample generated via classical molecular dynamics simulations and then optimized via DFT. The XC effects were considered in the adiabatic generalized gradient approximation (AGGA). The ELF obtained in Taioli *et al.* [65] has shown a good agreement with experiments. However, the orbital analysis of the ELF has not been performed in the *ab initio* framework.

In this work, we performed a detailed first-principles investigation of the electron scattering in liquid water both in the optical limit and for finite momentum transfer. Using an efficient iterative method based on LR-TDDFT and a linear combination of atomic orbitals (LCAO) incorporated in the mbpt-lcao code [66,67], we calculated the ELF of liquid water in a range of finite values of the momentum transfer. The electron–electron interactions were considered at the RPA level in the linear response, unlike in the work by Taioli *et al.* [65], who used the AGGA. RPA yields the correct asymptotic behaviour for the long-range interactions absent in AGGA [68]. The inelastic scattering cross sections, the IMFP and the electronic stopping power of electrons in water were then calculated from the ELF using analytical expressions [34,51]. Furthermore, we performed a detailed analysis of the contributions of molecular orbitals, chemical species and their pairs, as well as orbital angular momenta to the ELF. Additionally, we computed the cross sections for different molecular orbital channels which can benchmark semi-empirical calculations. Apart from the results presented in this article, we provide the data at https://doi.org/10.5061/dryad.d51c5b057 for the peruse in MC-TS simulations.

## 2. Methodology

### 2.1. Linear-response time-dependent density functional theory calculations with mbpt-lcao

The ELF is the fundamental quantity that defines the scattering properties of a material. It is defined as the imaginary part of the inverse macroscopic dielectric function *ϵ*_M_ [69]:

2.1 ELF(E,q)=Im[−1ϵM(E,q)],

which relates the external perturbation (potential) *V*^ext^ and the total potential *V*^tot^ acting in a system: *V*^ext^(*E*, **q**) = *ϵ*_M_(*E*, **q**)*V*^tot^(*E*, **q**).

In LR-TDDFT (see [70] for a broad overview), the main quantity that gives all the information about the response of a solid to an external perturbation *V*^ext^ is the microscopic dielectric function *ϵ*. The macroscopic dielectric function can be obtained from the microscopic one using the so-called macroscopic averaging, i.e. by averaging the microscopic quantities over all the unit cells, since macroscopic quantities slowly vary over the unit cell while microscopic ones vary rapidly [71]:

2.2 $$\epsilon_{M}(E,q)=\frac{1}{\epsilon_{G=0,G^{\prime }=0}^{-1}(E,q)}\neq \epsilon_{G=0,G^{\prime }=0}(E,q).$$

Here, **G, G′** are lattice vectors in the reciprocal space, which is more convenient to use when dealing with periodic systems. The differences between microscopic and averaged (macroscopic) fields are called the crystal local fields, or local field effects (LFE).

The inverse microscopic dielectric function is related to the interacting linear-response function $\chi_{{GG}^{\prime }}(E,q)=\delta n_{G}(E,q)/\delta V_{G^{\prime }}^{\mathrm{ext}}(E,q)$, an operator producing the induced density *δn* in response to a change of an external potential *δV*^ext^:

2.3 $$\epsilon_{GG^{\prime }}^{-1}(E,q)=\delta_{GG^{\prime }}+\upsilon_{GG^{\prime \prime }}(q)\chi_{{G^{\prime \prime }G}^{\prime }}(E,q),$$

where *ϒ*_**GG′**_(**q**) = 4*πδ*_**GG′**_/|**q** + **G**|^2^ is the Coulomb interaction matrix element between plane waves and *δ*_**GG′**_ is the Kronecker delta symbol. We adopt the repeated index sum convention in this section. Thus, the ELF in terms of the interacting response function reads

2.4 $$\text{ELF}(E,q)=-\frac{4\pi }{q^{2}}\text{Im}\chi_{G=0,G^{\prime }=0}(E,q).$$

In the KS formalism, the external potential is related to an effective potential *V*^eff^ (**r**, *t*) ≡ *V*^ext^(**r**, *t*) + *V*^Hxc^ (**r**, *t*), where Hxc stands for Hartree + XC potential—the relation generating a Dyson-type equation for the interacting *χ*_**GG**′_ (*E*, **q**) and non-interacting $\chi_{{GG}^{\prime }}^{0}(E,q)$ response functions [72]:

2.5 $$\chi_{{GG}^{\prime }}(E,q)=\chi_{{GG}^{\prime }}^{0}(E,q)+\chi_{{GG}^{\prime \prime }}^{0}(E,q)K^{G^{\prime \prime }G^{\prime \prime \prime }}(q)\chi_{G^{\prime \prime \prime }G^{\prime }}(E,q).$$

The crucial ingredient here is the interaction kernel *K*^**GG′**^(**q**), given by

2.6 $$K^{GG^{\prime }}(q)=\upsilon_{GG^{\prime }}(q)+f_{\mathrm{xc};GG^{\prime }}(q).$$

The XC kernel $f_{\mathrm{xc}}$ is the functional derivative of the time-dependent XC potential with respect to the time-dependent particle density and *ϒ*_**GG′**_ is the functional derivative of the Hartree potential with respect to the density. In this work, we calculated the LR-TDDFT interaction kernel *K*^**GG′**^(**q**) in the RPA approximation *K*^**GG′**^(**q**) = *ϒ*_**GG′**_. The XC effects are only taken into account in the ground state calculations. The RPA response function, only accounting for the Hartree component of the induced potentials, generally provides a good description of long-range screening [73].

The non-interacting response function appearing in equation (2.5) can be calculated as follows [71,74]:

2.7 $$\chi_{{GG}^{\prime }}^{0}(E,q)=\frac{1}{N_{k}}\sum_{n,m,k}\frac{\left(f_{n,k}-f_{m,k+q})U_{\mathrm{nm},k}^{G}(q){\bar{U}}_{\mathrm{nm},k}^{G^{\prime }}(q\right)}{E-(E_{m,k+q}-E_{n,k})+i\eta }.$$

Here, $f_{n,k}$ and $E_{n,k}$ are the occupation numbers and the energies of the corresponding KS eigenstates, *η* is a broadening constant and *N*_k_ is the number of *k*-points in the chosen Brillouin zone (BZ) sampling. $U_{\mathrm{nm}}^{G}(k,q)$ are the matrix elements of plane waves in the basis of KS eigenstates $\Psi_{n,k}(r)$

2.8 $$U_{\mathrm{nm},k}^{G}(q)=\frac{1}{\sqrt{V_{\mathrm{uc}}}}\int \Psi_{n,k}^{*}(r)e^{-i(G+q)r}\Psi_{m,k+q}(r)\;d^{3}r,$$

where *V*_uc_ is the unit cell volume.

The mbpt-lcao code uses an efficient iterative Krylov-subspace method to calculate the ELF (equation (2.4)). More details about the LR-TDDFT implementation in the mbpt-lcao code can be found in [66].

### 2.2. Partition of the electron energy loss function

The non-interacting response function $\chi_{{GG}^{\prime }}^{0}(E,q)$ has an explicit expression in terms of KS orbitals $\Psi_{n,k}(r)$, their eigenenergies $E_{n,k}$ and occupations $f_{n,k}$ (equations (2.7) and (2.8)). To analyse the contribution of different orbitals, as well as different species to the total ELF, we will express the ELF via the non-interacting response function. For this, it is convenient to rewrite equation (2.5) in the following form:

2.9 $$\chi =\chi^{0}{\left[\delta -K\chi^{0}\right]}^{-1}.$$

The operator [*δ* − *Kχ*^0^]^−1^converts the external potential $V_{G+q}^{\mathrm{ext}}(E)$ to an effective potential $V_{G+q}^{\mathrm{eff}}(E)$. In our case, $V_{G+q}^{\mathrm{ext}}(E)\equiv \delta_{G,0}$ and the effective potential $V_{G+q}^{\mathrm{eff}}(E)$ is computed by solving

2.10 $$[\delta_{GG^{\prime }}-K_{{GG}^{\prime \prime }}\chi_{G^{\prime \prime }G^{\prime }}^{0}(E,q)]V_{G^{\prime }+q}^{\mathrm{eff}}(E)=V_{G+q}^{\mathrm{ext}}(E).$$

In what is described in §2.1, the computation of the ELF is performed by applying the non-interacting response to the effective potential:

2.11 $$\text{ELF}(E,q)=-\frac{4\pi }{q^{2}}\text{Im}\chi_{{G=0,G}^{\prime }}^{0}(E,q)V_{G^{\prime }+q}^{\mathrm{eff}}(E).$$

Summing over the reciprocal lattice vectors **G**′, we get

2.12 $$\text{ELF}(E,q)=-\frac{4\pi }{q^{2}}\text{Im}\sum_{n,m,k}U_{\mathrm{nm},k}(q)D_{\mathrm{nm},k}(E,q),$$

where

2.13 $$U_{\mathrm{nm},k}(q)=\int \Psi_{n,k}^{*}(r)e^{-iqr}\Psi_{m,k+q}(r)\;d^{3}r$$

and

2.14 $$D_{\mathrm{nm},k}(E,q)=\sum_{G^{\prime }}(f_{n,k}-f_{m,k+q})\times \frac{\int \Psi_{m,k+q}^{*}(r)e^{i(G^{\prime }+q)r}\Psi_{n,k}(r)\;d^{3}r}{E-(E_{m,k+q}-E_{n,k})+i\eta }V_{G^{\prime }+q}^{\mathrm{eff}}(E).$$

Since equation (2.12) is linear in all the indices, we can split the ELF into different contributions. For example, the contributions of the electron–hole pairs can be defined as

2.15 $${\text{ELF}}_{\mathrm{nm},k}(E,q)=-\frac{4\pi }{q^{2}}\text{Im}\;[U_{\mathrm{nm},k}(q)D_{\mathrm{nm},k}(E,q)].\;$$

In practice, when the (atomistic) system is large and consists of many almost equal parts (water model), it is desirable to estimate the contributions of different (crystalline) orbitals defined by their energies $E_{n,k}$ to the total ELF(*E*, **q**). This is achieved by defining an occupied-energy differential ELF (DELF):

2.16 $${\text{DELF}}^{\mathrm{occ}}(E,q,E)=\sum_{n<m}\delta (E-E_{n,k}){\text{ELF}}_{\mathrm{nm},k}(E,q)+\sum_{m<n}\delta (E-E_{m,k+q}){\text{ELF}}_{\mathrm{nm},k}(E,q).$$

The contributions of different angular momenta, species or combination of these can be ‘tracked’ using an expansion of the KS orbitals in terms of the atomic orbitals:

2.17 $$\Psi_{n,k}(r)=\sum_{a}C_{n,k}^{a}\phi^{a}(r,k),$$

where $C_{n,k}^{a}$ are the LCAO coefficients and *ϕ*^*a*^(**r**, **k**) are the Bloch-symmetric atomic orbitals. The atomic orbital index *a* is connected to particular atoms, angular momenta, etc.

### 2.3. Inelastic scattering cross sections and stopping power from the energy loss function

As has been mentioned above, all the relevant quantities in the inelastic electron scattering can be calculated from the ELF [34,51]. According to the non-relativistic plane-wave Born approximation, the double-differential inelastic scattering cross section is defined as follows:

2.18 $$\frac{d^{2}\Sigma (E,q;T)}{dEdq}=\frac{1}{\pi a_{0}Tq}\text{ELF}(E,q),$$

where *T* is the incident electron kinetic energy, *a*_0_ is the Bohr radius. Note that here we used the magnitude *q* of the momentum transfer vector **q** since it is expected to be isotropic in liquid water (and our tests confirm this for our sample; see figure 13 in appendix A).

The single-differential cross section (SDCS) can be obtained from (2.18) by integrating over momentum transfer *q*:

2.19 $$\frac{d\Sigma (E;T)}{dE}=\frac{1}{\pi a_{0}T}\int_{q_{\min}(E;T)}^{q_{\max}(E;T)}\frac{\text{ELF}(E,q)}{q}\;dq,$$

where the limits $q_{\min/\max}(E;T)=\sqrt{2m}\left(\sqrt{T}\mp \sqrt{T-E}\right)$ come from momentum conservation (*m* is the electron rest mass).

The total inelastic cross section Σ (also called inverse IMFP) and the electronic (or collisional) stopping power *S*_e_ are defined as

2.20 $$\Sigma (T)=\int_{E_{\min}}^{E_{\max}(T)}\frac{d\Sigma (E;T)}{dE}\;dE\;$$

and

2.21 $$S_{e}(T)=-\frac{dT}{dx}=\int_{E_{\min}}^{E_{\max}(T)}E\frac{d\Sigma (E;T)}{dE}\;dE,$$

with the maximum energy loss by an electron with energy *T* being *E*_max_ = min[(*T* + *E*_gap_)/2, *T* − *E*_F_] for insulators, where *E*_gap_ is the energy gap of the target, and *E*_F_ is the Fermi energy; and the minimum defined as *E*_min_ = *E*_gap_ [47,51]. Note that this assumes that electronic excitation can only occur for energies larger than the gap, which acts as an effective threshold. However, recent studies have shown that this is possible also for energies below the gap [75,76]. Here, we go beyond the threshold, i.e. use the limit *E*_min_ = 0 in equations (2.20) and (2.21).

## 3. Numerical details

### 3.1. siesta single-point and mbpt-lcao calculations

The ground state KS orbitals of the water samples needed as a starting point for the LR-TDDFT calculations were obtained using the static DFT as implemented in the siesta code [77] using periodic boundary conditions. A 2 × 2 × 2 Monkhorst–Pack [78] *k*-point mesh was used in the siesta calculations. The XC functional in the local-density approximation (LDA) in the Ceperley–Alder form [79] was used. Norm-conserving Troullier–Martins [80] pseudopotentials were used to replace the core electrons. Basis sets of different sizes, i.e. single-, double- and triple-*ζ* polarized (SZP, DZP and TZP, respectively), with an energy shift of 20 meV were used in the test runs, and then the TZP basis set was chosen to perform the calculations of cross sections, IMFP and stopping power.

In the LR-TDDFT calculations, *q* cannot take values smaller than the distance between two *k*-points in the BZ. This distance can be estimated as 2*π*/(*n*_k_*a*), where *a* is the lattice constant and *n*_k_ is the number of *k*-points in a particular direction. For a BZ sampling of [3 × 3 × 3] *k*-points used in our calculations, we approximated the optical limit by $q_{0}=0.1\;a.u.$ We have calculated ELF for a total of 20 values of *q* in the interval [0.1 : 2.0] a.u. The resolution in energy loss was defined by Δ*E* = 0.15 eV and the broadening constant *η* = 0.3 eV (a sensible value must be ≥2Δ*E*).

### 3.2. Water samples

**Figure 1.**
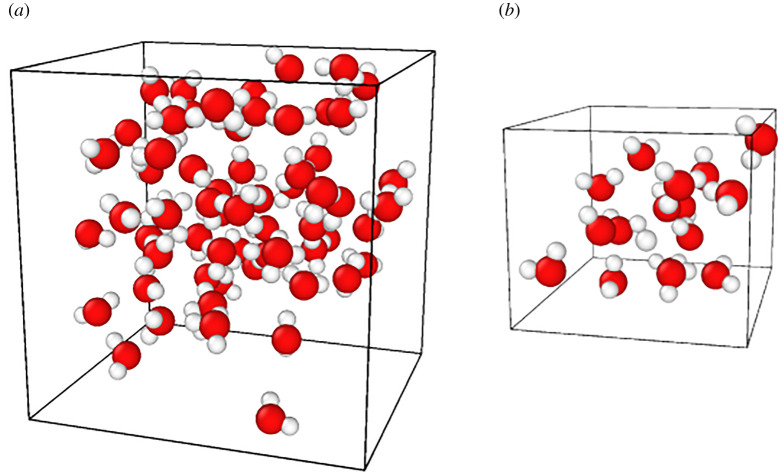
Unit cells of (*a*) PBE-64 and (*b*) PBE-16 water samples.

The results presented in §§4.1 and 4.2 were calculated for a water sample denoted as PBE-64 (referring to the XC functional and the number of water molecules) shown in figure 1*a*. The sample PBE-64 is composed of 64 water molecules which were initially randomly placed inside a cubic cell with a lattice constant *a* = 12.45 Å, giving a density of 0.995 g cm^−3^. The structure was equilibrated using Born–Oppenheimer molecular dynamics (BOMD) using the siesta code [77]. The BOMD simulations were performed for a total time of 2.5 ps at a temperature of 300 K in the NVT ensemble (Nosé thermostat) with default Nosé mass of 100 Ry fs^2^. A time step of 0.5 fs was used in the calculations. A DZP basis set of numerical atomic orbitals was used in the siesta BOMD simulations [81,82]. The cut-off radii of the first-*ζ* functions were defined by an energy shift of 20 meV. The second-*ζ* radii were defined by a split norm of 0.3. Soft confining potentials of 40 Ry with default inner radius of 0.9 were used in the basis-set generation [82]. The plane-wave cut-off for the real-space grid was defined by a mesh cut-off of 300 Ry. The self-consistency was controlled by a convergence parameter of 10^−3^ eV for the Hamiltonian matrix elements. The generalized gradient approximation (GGA) in the PBE [83] form was used to account for the XC effects.

The calculations presented in §§4.3 and 4.4 were performed for the water sample PBE-16 (figure 1*b*) composed of 16 water molecules to reduce the computational cost. The sample PBE-16 was optimized using BOMD with the same parameters as the sample PBE-64.

A few additional water samples of different sizes, atomic configurations and equilibrated with different XC functionals were used for testing purposes (see details in appendix A).

## 4. Results and discussion

### 4.1. Energy loss function

**Figure 2.**
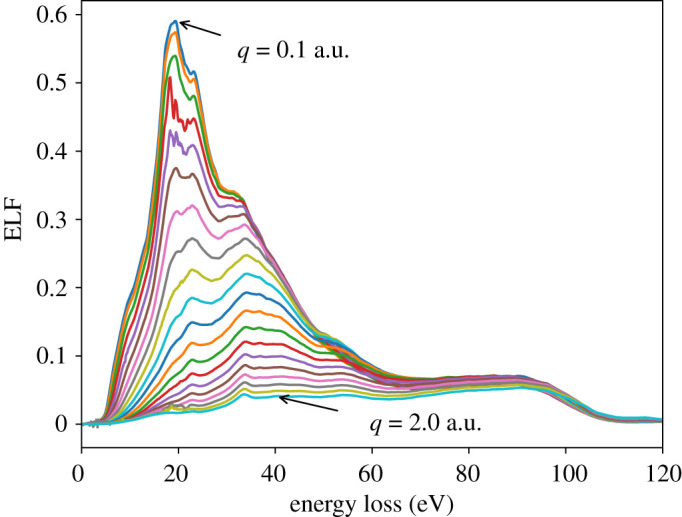
ELF of liquid water (a.u.) as a function of energy loss (eV) calculated with LR-TDDFT for *q* = [0.1 : 2.0] a.u. The PBE-64 water sample and TZP basis set were used in all calculations.

The ELF for different values of the momentum transfer is shown in figure 2 as a function of energy loss. The ELF exhibits a clear evolution for different values of the momentum transfer. At small *q*, a defined feature (i.e. a single maximum, accompanied by some shoulders) is clearly visible associated with the optical excitations sensitive to the optical band gap. For larger *q*, the energy loss involves larger wavevector excitations linked to the band structure of the system.

**Figure 3.**
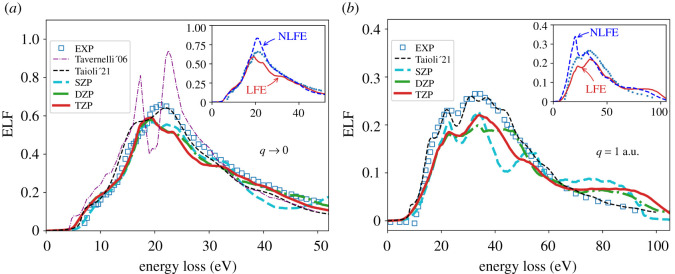
ELF of liquid water: (*a*) in the optical limit *q* = 0.1 a.u. and (*b*) for the momentum transfer *q* = 1.0 a.u. as a function of energy loss (eV) calculated with LR-TDDFT for PBE-64 water sample using different basis set sizes (SZP, DZP and TZP) in the siesta calculations as indicated for each line. LR-TDDFT results are compared to the experimental data obtained via IXS spectroscopy data [36] and to calculations by Tavernelli [64] (for *q* → 0) and Taioli *et al.* [65]. Insets show the ELF with and without local field effects (LFE and NLFE, respectively) compared to experiment.

Figure 3 shows the comparison of our results for the momentum transfer of 0.1 and 1 a.u. with inelastic X-ray scattering (IXS) experimental data [36]. A convergence test for the ELF with respect to the basis set size is presented as well. The DZP and TZP results for the ELF at q=0.1 a.u. are almost identical. At q=1.0 a.u., the DZP and TZP slightly vary, with TZP closer reproducing main experimental features around the maximum of the ELF. Main panels show the results obtained including the LFE, while the insets compare the results obtained with and without the LFE to the experimental data.

In the optical limit (figure 3*a*), the tails of the ELF from IXS experiments are well reproduced by our well-converged (TZP) calculations. The main peak is slightly underestimated and appears slightly shifted to lower energy loss values as compared to the experiment. The results of Tavernelli [64] obtained with real-time TDDFT (RT-TDDFT) are also shown in figure 3*a* and, as has been mentioned before, the RT-TDDFT ELF shows two maxima with a minimum located at similar energy loss values as the experimental maximum.

Recent calculations by Taioli *et al.* [65] obtained with LR-TDDFT (including XC effects in the AGGA) are also shown in figure 3. The main peak is captured well by Taioli *et al.* [65]; however, a feature at *E* ≈ 15 eV is higher than in the experimental ELF, and the main peak shows two features (although much less prominent) similar to the results of Tavernelli. Thus, our RPA results better reproduce both the lower and higher energy-loss side of the main peak, while the AGGA results from Taioli *et al.* [65] better reproduce the main peak. Often, RPA with LFE is able to reproduce fairly well the energy loss spectra at *q* = 0 [84]. AGGA generally brings an improvement upon RPA in finite systems [85]. However, for extended non-metallic systems, the XC-kernel effect vanishes due to the absence of the long-range (1/*r*) decay [85–87] and thus AGGA yields a relatively small correction to the RPA results [88,89]. The inclusion of the LFE, however, plays a significant role in the correct description of the electron energy loss [88], as proven by our results.

At finite value of the momentum *q* = 1 a.u., positions of experimental peaks and their widths are well captured by our calculations (figure 3*b*). However, the height is slightly underestimated and there is a plateau at high energy loss in the calculated ELF which is not present in the experimental data. Taioli *et al.* [65] do not get such plateau in their calculations and they capture the height and width of the peaks quite accurately. Overall, this comparison clearly confirms, in a TDDFT framework and considering the system beyond an electron gas as done in optical data models for TS calculations, that considering XC effects is more important for finite momentum transfer, as already anticipated by previous works in optical data models [41].

Apart from intensities and position of the peaks, one should also discuss the ‘fall off’ on the sides of the main structure. The extension algorithms used in many MC TS codes (e.g. in Geant4-DNA [23]) do not account for the momentum broadening of the ELF being largely based on the early Ritchie model(s) and the Ashley model, which exhibit a much steeper fall below the maximum. However, more recent MC TS codes have included momentum broadening either empirically [51] or via the Mermin dielectric function [90].

In the insets to figure 3, we compare the ELF obtained with the LFE, which is the same as the TZP results of the main panels, and without LFE (i.e. NLFE). In an inhomogeneous and polarizable system, on a microscopic scale, the LFE imply that the matrix *ε*_**GG**′_ has non-zero off-diagonal elements. In practical terms, it means that the Coulomb interactions between the electrons of the system (i.e. the Coulomb kernel, equation (2.6)) are included. The LFE are stronger when the inhomogeneity of the system is larger. Although, if the microscopic polarizability of the inhomogeneous system is small, the LFE are small. For *q* → 0, the LFE only change the height of the main feature (inset to figure 3*a*), maintaining to a large extent the overall shape of the NLFE result. Practically, the LFE suppress the absorption, due to the induced classical depolarization potential. LFE are expected to give a small contribution in situations and systems with a smoothly varying electronic density. On the contrary, the LFE give a more sizable contribution for larger wavevectors as seen in the inset to figure 3*b*.

### 4.2. Inelastic scattering cross sections and electronic stopping power

Understanding the energy (and angle) distribution of secondary electrons is fundamental for characterizing the physical step of radiation bio-damage. Indeed, such energy will determine not only the specific processes by which such electrons will interact with the biological molecules in the water solvent, but also how close bond-breaking events, possibly occurring at nucleobase pairs or at the sugar–phosphate chain, occur (clustering). Depending on the clustering of these events, the damage may be irreparable. Here, we present the calculated single-differential as well as total inelastic scattering cross sections and compare our results with the calculations from the dielectric formalism. The IMFP and the stopping power are also compared to the results from the default, Ioannina and CP100 models as implemented in Geant4-DNA.

**Figure 4.**
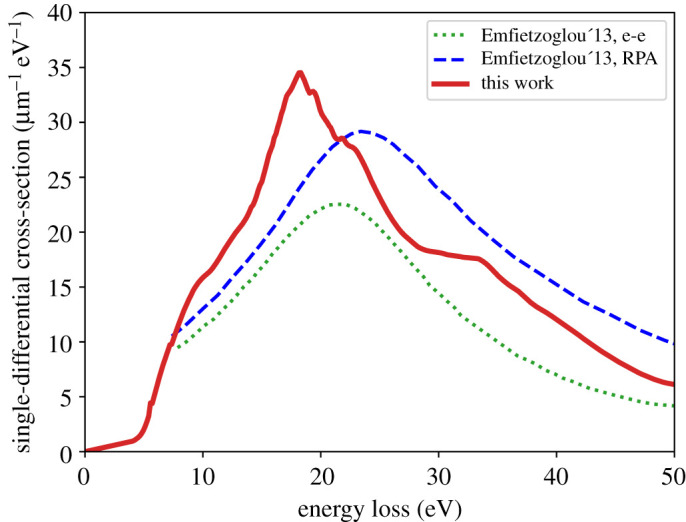
Single-differential cross section (equation (2.19)) for incident electron kinetic energy *T* = 100 eV. Results from Emfietzoglou *et al.* [51] are shown for comparison.

Figure 4 shows the SDCS calculated using equation (2.19) for the electron kinetic energy *T* = 100 eV in comparison with different approximations of the Emfietzoglou model [51]. The label ‘e-e’ in Emfietzoglou *et al.* [51] results stands for the semi-empirical electron–electron dielectric function representing an XC corrected screened interaction between the incident and struck electrons. The label ‘RPA’ stands for the random-phase approximation within the Lindhard formalism for dielectric function obtained under the plasmon-pole approximation for a homogeneous electron gas. Further details can be found in Emfietzoglou *et al.* [51]. The maximum of SDCS obtained in this work is shifted to lower values of the energy loss when compared with the semi-empirical results. Overall, the agreement is qualitative. The SDCS for electron kinetic energies of 500 eV, 1 keV and 5 keV are given in appendix A (figure 14).

**Figure 5.**
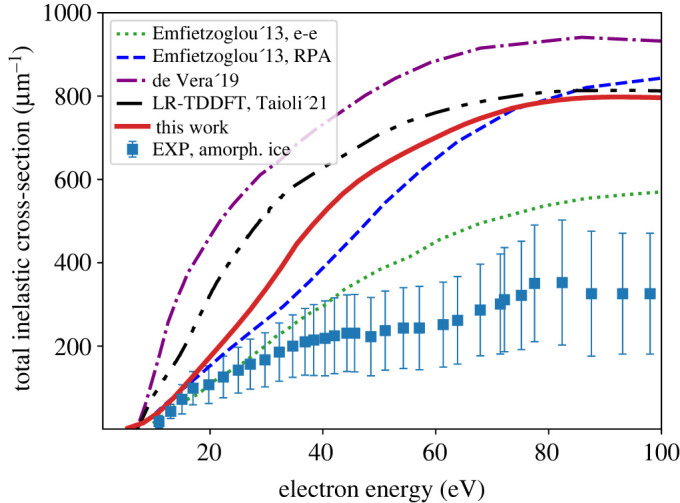
Total inelastic scattering cross section (equation (2.20)) as a function of the electron incident kinetic energy compared to experimental data for amorphous ice (EXP, amorph. ice) [91] and calculations by Emfietzoglou *et al.* [51], de Vera *et al.* [92] and Taioli *et al.* [65] (the sum of the excitation and ionization cross sections presented in fig. 3 of [65]).

The total inelastic cross section is shown in figure 5 and compared to the results of Emfietzoglou *et al.* [51], de Vera *et al.* [92] and Taioli *et al.* [65], as well as with experimental data for amorphous ice. Here, the total cross section is expressed in the units of inverse length, i.e. the conventional units of length squared multiplied by the number density of the target atoms [51]. Our result agrees with the RPA model of Emfietzoglou *et al.* being slightly higher at intermediate electron energies. Both RPA calculations, our LR-TDDFT and the Emfietzoglou model, converge to the experimental curve only at energies below 20 eV. Recent LR-TDDFT results from Taioli *et al.* [65] are slightly higher than ours, but become similar at energies above 80 eV.

**Figure 6.**
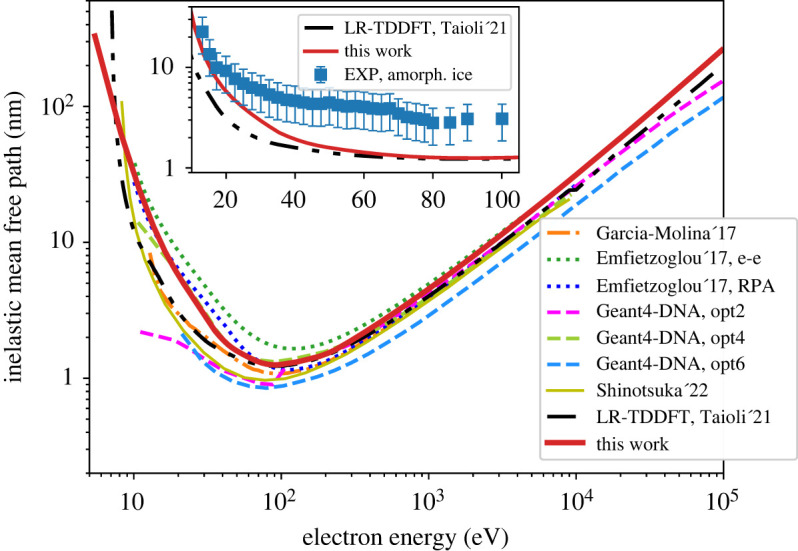
Inelastic mean free path as a function of the electron incident kinetic energy compared to calculations by Garcia-Molina *et al.* [47] (dash-dotted line), Emfietzoglou *et al.* [39] (dotted lines), Geant4-DNA options 2, 4 and 6 [23] (dashed lines), Shinotsuka *et al.* [93] and Taioli *et al.* [65] (dash-dot-dotted line, calculated as the inverse of the total cross section shown in figure 5). The inset shows the comparison of the LR-TDDFT results from this work and from Taioli *et al.* [65] with experimental data for amorphous ice [91].

The IMFP obtained in this work is shown in figure 6 and compared to the semi-empirical calculations [39,47], to the LR-TDDFT results from Taioli *et al.* [65], and to three Geant4-DNA constructors (the default, the Ioannina and the CPA100 models, denoted as opt2, opt4 and opt6) [23]. A more recent semi-empirical result of Shinotsuka *et al.* [93] obtained using the relativistic full Penn algorithm that includes the correction of the bandgap effect in water is also shown in figure 6. Our results quantitatively agree with the RPA model of Emfietzoglou *et al.* [39] in the whole energy range and with the IMFP obtained by Taioli *et al.* [65] at intermediate energies (from 100 eV to 10 keV). The data of Shinotsuka *et al.* [93] are below our IMFP and resemble more the shape of the results of Taioli *et al.* [65], except for the region around the minimum. The inset of figure 6 shows the comparison of the LR-TDDFT results from this work and from Taioli *et al.* [65] with the experimental data for amorphous ice below 100 eV. Both calculated results agree well at energies above 50 eV. Below this energy, the results of this work are closer to the experimental data than the ones from Taioli *et al.* [65].

In the Geant4-DNA default option (opt2), the total and inelastic cross sections for weakly bound electrons are calculated from the energy- and momentum-dependent complex dielectric function within the first Born approximation. In particular, the optical data model of Emfietzoglou *et al.* [94,95] is used, where the frequency dependence of the dielectric function at *q* = 0 is obtained by fitting experiments for both the real and imaginary part of the dielectric function, using a superposition of Drude-type functions with adjustable coefficients. A partitioning of the ELF to the electronic absorption channels at *q* = 0, proportional to the optical oscillator strength, enables the calculation of the cross sections for each individual excitation and ionization channel. The extension to the whole Bethe surface is made by semi-empirical dispersion relations for the Drude coefficients. Below a few hundred eV, where the first Born approximation is not applicable, a kinematic Coulomb-field correction and Mott-like XC terms are used [95]. For ionization of the O K-shell, total and differential cross sections are calculated analytically using the binary-encounter-approximation with exchange model (BEAX), an atomic model which depends only on the mean kinetic energy, the binding energy, the occupation number of the electrons, and where the deflection angle is determined from the kinematics of the binary collision, thus referring to sole vapour data.

In the Ioannina model [96,97], two problems appear in the default option, e.g. a brute-force truncation of the Drude function violating the *f*-sum rule and the consequent complexity in deriving *ϵ*_*R*_(*E*, *q*) from *ϵ*_*I*_(*E*, *q*) via Kramers–Kronig relations [23] are overcome via an algorithm which redistributes *ϵ*_*I*_(*E*, *q* = 0) to the individual inelastic channels in a *f*-sum rule constrained and physically motivated manner. Below a few hundred eV, more accurate ionization cross sections, especially at energies near the binding energy, are obtained via methodological improvements of the Coulomb and Mott corrections. In the CPA100 models [98], excitation cross sections are calculated in the first Born approximation using the optical data model by Dingfelder *et al.* [33], which is also based on a Drude-function representation of *ϵ*(*E*, *q*) but uses a different parametrization. The ionization cross sections are calculated via the binary-encounter-Bethe (BEB) atomic model.

As no international recommendations exist yet for the mean free path, the only conclusion we can draw from the comparison between our results and the Geant4-DNA (figure 6) is that our first-principles result seems to well reproduce the Geant4-DNA opt4 at energies up to 1 keV. Above this energy, our result is slightly higher than opt2, opt4 and opt6. For the whole energy range, the IMFP from opt6 appears to be the lowest because of the larger inelastic cross sections in the 10 eV–10 keV range, as a consequence of using an atomic ionization model with the absence of screening [23]. The curve for opt2 shifts suddenly, through a clearly visible step, below 200 eV because of the activation of vibrational excitations, which reduce additional energy losses and thus reduce the IMFP. In opt4, excitations are strongly enhanced compared to ionization, the latter decreasing only moderately, which results in higher *W* values (the average energy to produce an ion pair) and smaller penetration distances [96]. Since XC effects mostly affect the results for *q* ≠ 0, it is expected that XC corrections to the RPA will mostly influence the IMFP at low energies, where large-angle scattering collisions (*q* ≠ 0) become important [39,51]. Indeed, as the comparison in the inset of figure 6 shows, the RPA result of this work differs from the AGGA results of Taioli *et al.* [65] only at energies below 50 eV. However, the inclusion of exchange and correlation does not improve the RPA result with respect to the experimental data.

**Figure 7.**
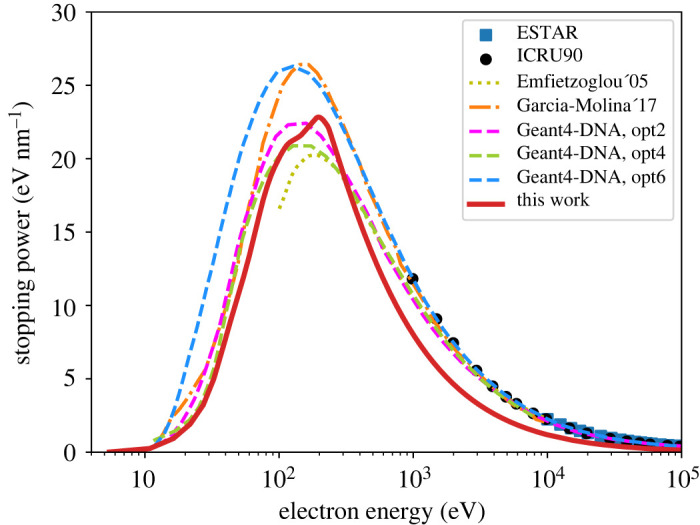
Electronic stopping power (equation (2.21)) as a function of the electron kinetic energy. Our results are compared to ESTAR [99] and ICRU90 [100] data, as well as calculations by Emfietzoglou *et al.* [34] (dotted line), Garcia-Molina *et al.* [47] (dash-dotted line) and Geant4-DNA options 2, 4 and 6 [23] (dashed lines).

The stopping power from LR-TDDFT is shown in figure 7 in comparison with the semi-empirical calculation [34,47], three Geant4-DNA options, and data from ICRU and ESTAR. As was the case for IMFP, our result is closer to opt4, until a few hundreds of eV, while at higher energies our stopping power is considerably smaller than the rest of the data presented. However, our stopping recovers the correct limit at the highest energies (10–100 MeV).

### 4.3. Analysis of different contributions to the electron energy loss function

Following the partition method described in §2.2, we calculated the ELF separated on contributions from different species, angular momenta and atomic pairs.

**Figure 8.**
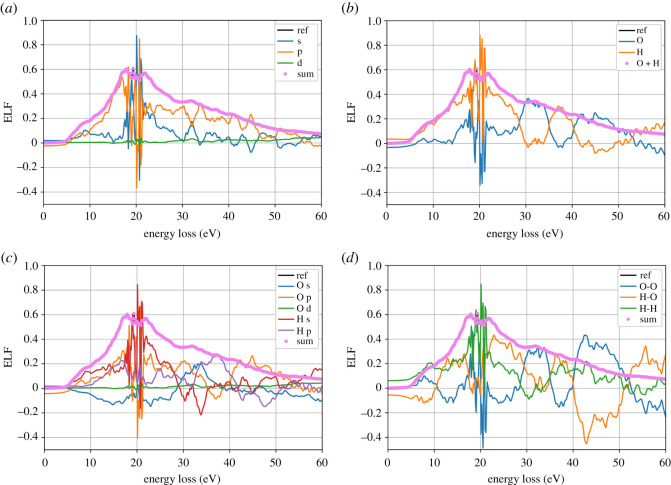
Contributions of (*a*) different angular momenta, (*b*) species, (*c*) angular momenta of different species and (*d*) pairs of species, to the total ELF (q=0.1 a.u.) labelled as ‘ref’.

Figure 8*a* shows the contribution of orbital angular momenta *s*, *p* and *d* of all the atoms to the total ELF. Clearly, *p*-orbitals play a major role in forming the slopes of the ELF with a smaller contribution from *s*-orbitals. The maximum of the total ELF comes from a complicated interplay between the *s*- and *p*-levels, for which the ELF oscillates in an incoherent way. The results are validated by summing up all the contributions which add up to the total ELF.

Figure 8*b* shows the contribution of all the oxygen atoms and all the hydrogen atoms to the total ELF. Below the maximum, the total ELF mostly comes from the hydrogen atoms. At the maximum, hydrogen atoms contribute more to the total ELF, while both curves again oscillate in opposite phases. Above the maximum, the two contributions oscillate resulting in a rather smooth total slope.

A detailed analysis, separating *s*, *p* and *d* orbitals of hydrogen and oxygen (figure 8*c*), shows that the main contribution to the ELF maximum is from the hydrogen *s*-levels and oxygen *p*-levels. Oxygen *s*-orbitals mostly contribute negatively. A significant contribution comes from the hydrogen *p*-orbital, indicating the excitation of the hydrogen electrons which populate the *p*-shell. Oxygen *d*-shell remains mostly unpopulated.

The contributions of species pairs are shown in figure 8*d*. Again, hydrogen plays the main role at low energy loss as H–H and H–O pairs. At the maximum of ELF, both H–O and O–O pairs contribute negatively to the total ELF. Similarly to the case of different species, there are incoherent oscillations in ELF above 25 eV for O–O and H–O pairs. However, in this case, the two contributions oscillate around zero cancelling each other. Thus, at high energy loss, the total ELF is mostly due to the H–H pairs.

### 4.4. Energy loss function and single-differential cross section for each molecular orbital

When constructing the Drude-type dielectric response function in semi-empirical methods, the continuum in the fitting procedure is usually represented by the outer shells of the water molecule [69,101]. Thus, the analysis of the ELF, and consequently the cross-sections, for each molecular orbital of water can be of interest for the MC track structure community for benchmarking of the semi-empirical models.

**Figure 9.**
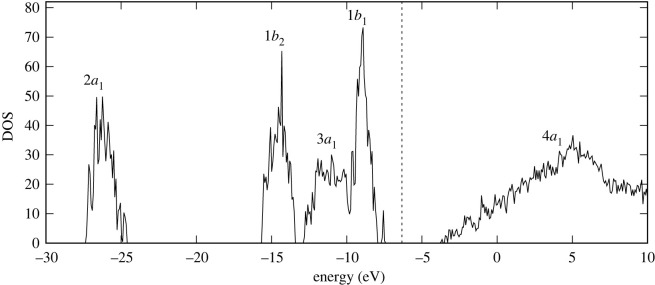
Electronic density of states of liquid water obtained within DFT. Vertical dashed line shows the Fermi level at −6.3 eV.

Here, we calculate the ELF for each occupied molecular orbital of the water molecule, i.e. the orbitals 2*a*_1_, 1*b*_2_, 3*a*_1_ and 1*b*_1_ [102]. In liquid water, the bands of crystal orbitals correspond to different symmetries of an isolated molecule. The bands can be seen in the electronic density of states (DOS) of liquid water sample shown in figure 9 as a function of energy which we calculated using the DFT implementation of the siesta code [77]. The binding energies of the four occupied orbitals of water are known from photoemission experiments. For liquid water, the binding energies are 30.90 eV for 2*a*_1_, 17.34 eV for 1*b*_2_, 13.50 eV for 3*a*_1_ and 11.16 eV for 1*b*_1_ [103]. Our results are slightly higher than experimental data, which is expected from the DFT method. However, our DOS is in good agreement with other DFT calculations [104].

Each feature in the DOS (figure 9) is labelled with the symmetry group corresponding to the isolated molecule. The DOS includes four outer occupied bands with symmetries 2*a*_1_, 1*b*_2_, 3*a*_1_ and 1*b*_1_ and one unoccupied band with the symmetry 4*a*_1_. For the ELF calculations, we only considered the occupied orbitals, i.e. the ones located below the Fermi level.

**Figure 10.**
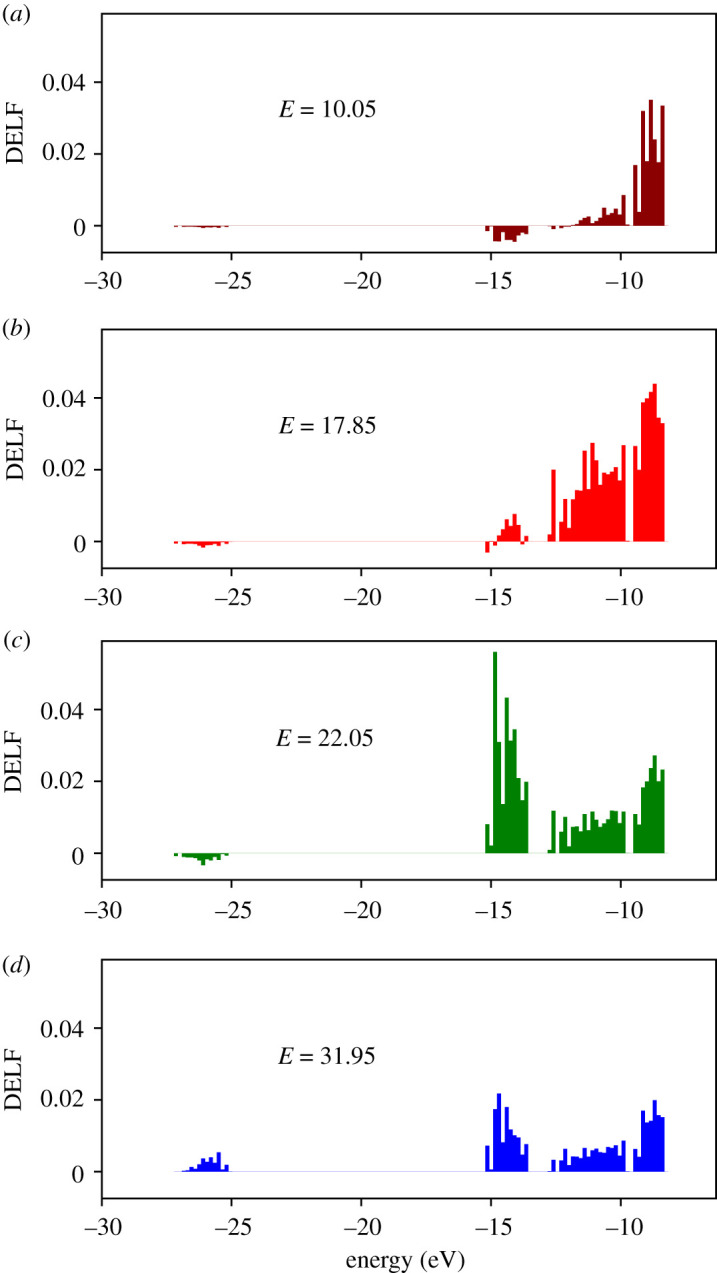
Contributions of different occupied states to the total ELF (*q* = 0.1) resolved in energy shown on panels (*a*–*d*) for the energy loss *E* values 10.05, 17.85, 22.05 and 31.95 eV, respectively.

Since we cannot directly obtain the ELF for each molecular orbital from LR-TDDFT calculations, we sum up the values of ELF for all occupied crystal orbitals within the energy window corresponding to each symmetry. Figure 10 shows the contributions of occupied states to the total ELF resolved in energy in the optical limit for four selected values of the energy loss *E* = 10.05, 17.85, 22.05 and 31.95 eV, corresponding to the regions below, around and above the maximum of ELF (figure 3*a*). One can clearly distinguish four energy windows in which the ELF has non-zero values that can be directly correlated with the DOS (figure 9). Thus, summing up the ELF of each crystal orbital in each of the energy windows, we obtained the ELF for four molecular orbitals of water.

**Figure 11.**
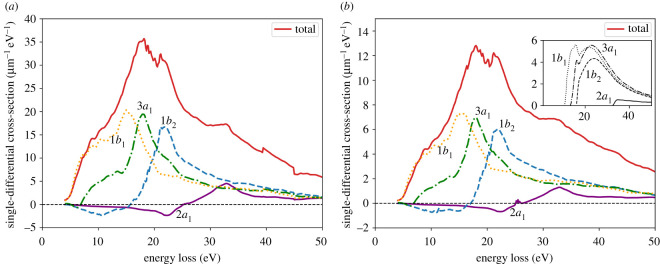
Single-differential cross section for each symmetry of a water molecule. Incident electron kinetic energy is (*a*) *T* = 100 eV and (*b*) *T* = 500 eV. The inset in (*b*) shows the results from [33].

**Figure 13.**
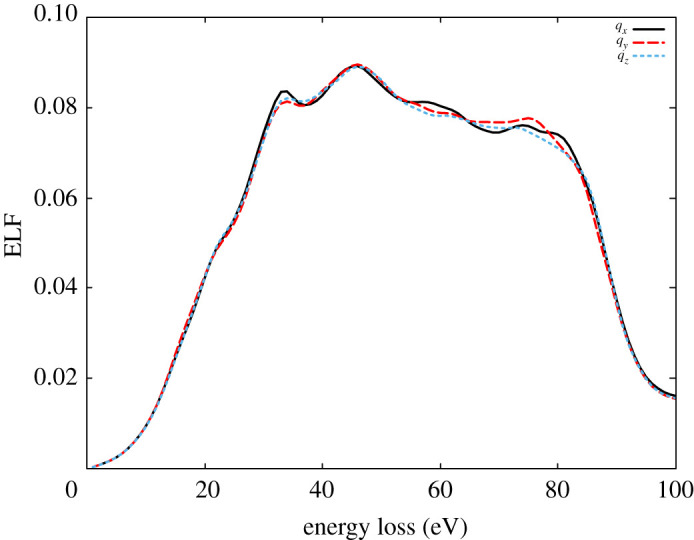
ELF of liquid water (sample PBE-64) for q=1.5 a.u. as a function of energy loss (eV) calculated with LR-TDDFT for different directions of the momentum transfer: *q*_*x*_, *q*_*y*_ and *q*_*z*_.

**Figure 14.**
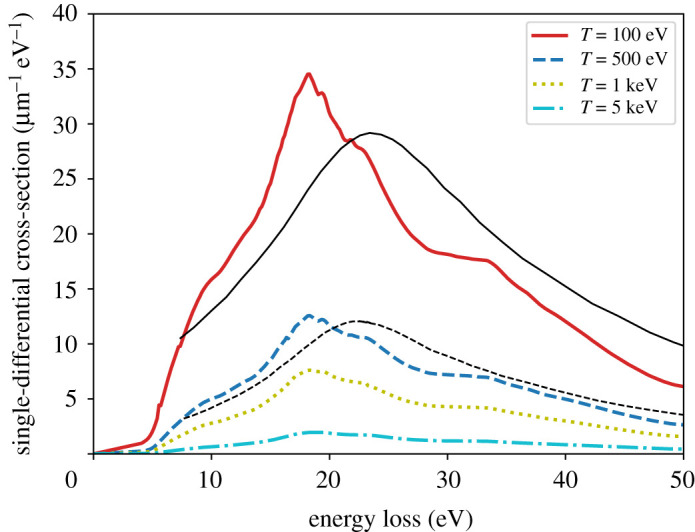
The SDCS for the electron kinetic energies of 100 eV, 500 eV, 1 keV and 5 keV as a function of energy loss. Thin black lines show the RPA results from Emfietzoglou *et al.* [51] for electron energies of 100 eV (solid black line) and 500 eV (dashed black line).

We repeated the calculations described above for the same values of the momentum transfer *q* as in figure 2 to obtain the partitioned ELF in the whole Bethe surface (*E*, *q*). This allowed us to compute the cross sections corresponding to each molecular orbital using equation (2.19). As an example, figure 11 shows the SDCS for the incident electron with kinetic energies *T* = 100 eV and 500 eV in water sample with 16 molecules. Each molecular orbital is observed to be responsible for a certain feature in the total SDCS. The partitioning also looks very similar at both electron incident energies. The cross section for energy losses below 10 eV is almost entirely due to the contribution from the highest occupied molecular orbital (HOMO) 1*b*_1_. Deeper shells contribute at higher energy losses. The orbital 2*a*_1_ has a minor contribution only at high energy losses. The observed behaviour is in a qualitative agreement with available data from the dielectric formalism within the first Born approximation [33,69] (see inset to figure 11*b*).

## 5. Conclusion

In summary, in this work, we computed several quantities important for the description of inelastic scattering of electrons in liquid water using a linear-response formulation of TDDFT. A good agreement with experimental data was obtained for ELF in the optical limit as well as at finite values of the momentum transfer. We thoroughly tested our results for dependence on the system size and the choice of the DFT parameters.

Additionally, we provided a detailed analysis of the ELF in the optical limit in terms of contributions from different species, species pairs and orbital angular momenta.

Furthermore, we computed the SDCS, total inelastic cross section, IMFP and the electronic stopping power from the ELF. Our results are in a good agreement with the semi-empirical calculations. Thus, LR-TDDFT offers an alternative method to the standard semi-empirical calculations and provides useful input for more detailed MC-TS simulations. It is envisioned that the investigated quantities have the potential to be of direct use in open source TS codes like Geant4-DNA. In particular, the decomposition of the cross sections on different molecular orbital channels, calculated *ab initio* for the first time in this work, can be used as a benchmarking for semi-empirical models.

## Figures and Tables

**Figure 12.**
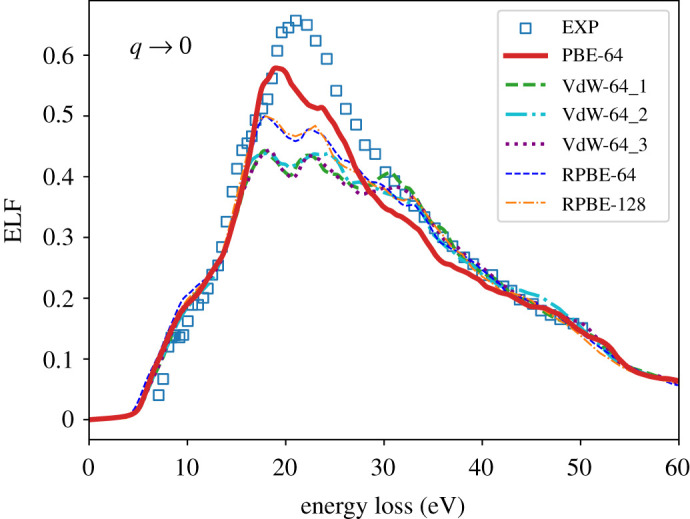
ELF of liquid water in the optical limit (*q* → 0) as a function of energy loss (eV) calculated with LR-TDDFT for different water structures (see text for details) with the broadening constant *η* = 0.3 eV and the momentum transfer q=0.1 a.u. The DZP basis set with an energy shift of 20 meV is used in the siesta calculations. IXS data (*q* = 0) is from Hayashi *et al.* [36].

## Data Availability

Data files with the IMFP, the electronic stopping power and the SDCS for kinetic energies of 100 eV, 500 eV, 1 keV and 5 keV are provided in the Dryad Digital Repository: https://doi.org/10.5061/dryad.d51c5b057 [108].

## Appendix A. Test results and additional data for single-differential cross section

To check the convergence of our results with respect to the particular configuration of the water molecules in the sample, we have calculated the ELF for several different samples. One of the samples with the initial configuration of PBE-64 was equilibrated with the van der Waals (DRSLL) [105,106] XC functional in the BOMD simulation. Three snapshots were chosen, after 2.7 ps, 5.0 ps and 6.8 ps of the BOMD, denoted with subscripts VdW-64_1,2,3 to test the convergence of the LR-TDDFT results with the sample configuration. The sample RPBE-64 (RPBE-128) contains 64 (128) molecules in a cubic unit cell with the lattice constant a=12.43 (15.66) Å to correspond to the experimental water density at 300 K and 1 bar. The SCF convergence threshold during the MD was st to 2 × 10^−7^. The equilibration was performed with BOMD of the CP2K code [107], with RPBE-D3 XC functional and a TZV2P basis set at 300 K in the NVT ensemble (Nosé-Hoover-chains thermostat) for 10 ps. A larger sample with 128 molecules was used to test the dependence of our results on the system size.

The tests have shown that the ELF is similar for all the structures and that it does not depend on the system size or a particular snapshot configuration among the ones generated as described, once properly equilibrated (figure 12). The three different VdW snapshots give practically the same results. The same is true for both RPBE samples, with 64 and 128 molecules, the use of which leads to the same results for the ELF. Overall, the tests show that the ELF depends on neither the size of the sample used in the calculations, nor the exact configuration of the water molecules in the sample. The choice of the XC functional, however, affects the ELF results in the peak area, with the PBE functional giving the closest result to the experimental data. For this reason, we chose to perform all the calculations for the cross sections and stopping power using the ELF obtained with the PBE-64 structure.

The use of the scalar value of the momentum transfer is justified by the fact that the ELF is isotropic, i.e. it is very similar for all the directions of the momentum transfer vector, as can be seen in figure 13.

Figure 14 presents the SDCS for incident electron kinetic energies of 100 eV, 500 eV, 1 keV and 5 keV compared to available results from Emfietzoglou *et al.* [51] for energies of 100 and 500 eV.
